# Access, utilization, and awareness for clinical genetic testing in autism spectrum disorder in Sweden: A survey study

**DOI:** 10.1177/13623613211066130

**Published:** 2021-12-28

**Authors:** Anna Hellquist, Kristiina Tammimies

**Affiliations:** 1 Center of Neurodevelopmental Disorders (KIND), Centre for Psychiatry Research; Department of Women’s and Children’s Health, Karolinska Institutet and Child and Adolescent Psychiatry, Stockholm Health Care Services, Stockholm County Council, Stockholm, Sweden; 2 Astrid Lindgren Children’s Hospital, Karolinska University Hospital, Region Stockholm, Sweden

**Keywords:** access to services, genetic counseling, genetic testing

## Abstract

Clinical genetic testing is recommended for individuals diagnosed with autism spectrum disorder. There are only a few reports of how these recommendations are followed and especially missing for European countries. We aimed to analyze the rate of access, utilization, and awareness of clinical genetic testing among autistic individuals in Sweden through online surveys targeting parents with at least one autistic child and autistic adolescents (from 15 years) and adults. In total, 868 parents of autistic children and 213 autistic adolescents or adults completed the survey. Only 9.1% (*n* = 79) of parents and 2.8% (*n* = 6) of autistic adolescents/adults reported having received a referral for clinical genetic testing after autism spectrum disorder diagnosis. The autistic children offered a referral were younger at diagnosis (*p* < 0.001) and more likely to have an additional neurodevelopmental diagnosis (*p* < 0.01), including intellectual disability (*p* < 0.001) or a language disorder (*p* < 0.001). Genetic counseling was provided to less than half of the families that were referred for clinical genetic testing. Finally, we report that both respondent groups preferred to be informed by written text and an expert in genetics about clinical genetic testing. This study highlights a lack of awareness and access to clinical genetic testing after autism spectrum disorder diagnosis in Sweden and demonstrates the need for additional studies on how clinical guidelines for genetic testing are followed in different countries.

**Lay abstract:**

Several medical professional societies recommend clinical genetic testing for autistic individuals as many genetic conditions are linked to autism. However, it is unclear to what extent autistic individuals and parents of autistic children are offered clinical genetic testing. We conducted a community-based survey to estimate the access, utilization, and awareness for clinical genetic testing in Sweden. In total, 868 parents of autistic children and 213 autistic adolescents or adults participated as respondents. The referral rate for clinical genetic testing after autism spectrum disorder diagnosis was low, with only 9.1% for the autistic children as reported by their parents and 2.8% for autistic adolescents/adults. The autistic children who got referrals were more likely to have intellectual disability and language disorder. We also report that awareness of the clinical genetic testing possibility was low in both respondent groups. We also highlight preferred communication means and needs for information before clinical genetic testing. Our results show that utilization and access are low in Sweden, and more studies should be conducted to report these rates in different countries to analyze the effects of clinical genetic testing on healthcare for autistic individuals. Our results highlight the most important information for the families and how the information should be communicated prior to clinical genetic testing.

## Introduction

Autism spectrum disorder (ASD) is a heterogeneous neurodevelopmental disorder (NDD) with a strong genetic basis (Lord et al., 2020). The global prevalence estimate of ASD is approximately 0.62% (1 in 160 children); however, high variability in the estimates has been shown across regions and countries (Chiarotti & Venerosi, 2020). In Sweden, there has been a detailed mapping of the prevalence, especially for the Stockholm Region. In 2017, the ASD prevalence estimates in Stockholm were 1.4% for children between 0 and 12 years and 3.0% for the age span between 12 and 18 years, and 2.4% for 18 and 24 years (Center for Epidemiology and Community Medicine, Region Stockholm, 2017).

As there is no medical or biomarker test for ASD, the diagnostic process is based on objective assessments and interviews by the healthcare staff. In Sweden, the diagnostic procedure for ASD can vary depending on the healthcare region and the clinic. However, in general, it should be performed by a medical doctor, who is responsible for the medical examination, and a psychologist who performs the needed cognitive and behavioral tests and diagnostic interviews.

As multiple genetic syndromes and genomic disorders are underlying ASD, clinical genetic testing (CGT) is recommended to autistic individuals by several medical professional societies (Schaefer et al., 2013), including The Swedish Pediatric Association (The Swedish Neuropediatric Section of The Society for Swedish Pediatricians, 2019). The most common CGT methods after ASD diagnosis are targeted testing for Fragile X syndrome (Moeschler et al., 2014), and copy number variant (CNV) screens using chromosomal microarray (CMA) (Miller et al., 2010; Schaefer et al., 2013). However, recently a consensus statement concluded that whole-exome sequencing (WES) should be used as the first-tier CGT for autistic individuals (Srivastava et al., 2019). Furthermore, the American College of Medical Genetics and Genomics (ACMG) also recommended that WES, or whole genome sequencing (WGS), should be considered as the first- or second-tier for children with congenital anomalies (CAs), developmental delay (DD), or intellectual disability (ID), given the higher diagnostic yield (Manickam et al., 2021). The estimates of molecular diagnostic yield (number of the test giving positive results) vary for ASD; approximately 8%–15% of the tested autistic individuals have a pathogenic CNV with CMA and a 8%–25% yield from WES (Srivastava et al., 2019; Tammimies et al., 2015).

The Swedish Pediatric Association recommends testing for Fragile X syndrome only for autistic children with comorbid ID (The Swedish Neuropediatric Section of The Society for Swedish Pediatricians, 2019). Similarly, CMA should be offered to autistic children with ID, uncertain developmental disability, malformations, dysmorphic features, and/or consanguinity. CGT for ASD in Sweden is performed by one of the university hospital clinical genetics departments located in each of the six larger healthcare regions in Sweden after a referral from, for instance, a pediatrician, a medical doctor in the child- and adolescent psychiatric clinic or neurologists (The Swedish Neuropediatric Section of The Society for Swedish Pediatricians, 2019).

Based on survey information and health records, between 16.5% and 60% of autistic children or adults had undergone CGT in the United States (Cuccaro et al., 2014; Harris et al., 2020; Kiely et al., 2016; Moreno-De-Luca et al., 2020; Vande Wydeven et al., 2012; Zhao et al., 2019a). Also, over half of child and adolescent psychiatrists in the United States reported that they had ordered a genetic test in the practice within the last 12 months, and the majority of these were related to ASD (Soda et al., 2021). In Europe, there are relatively few reports on the utilization and access to CGT. A Spanish study showed that out of 130 families with at least one autistic child, 30% had visited a genetics service, and of these, 13% had undergone CMA (Codina-Solà et al., 2017). In France, up to 62% of families have undergone CGT after ASD diagnosis (Amiet et al., 2014).

As there are no earlier published reports of the utilization of CGT for ASD in Sweden, it is unclear to what extent the international and national guidelines and recommendations are implemented and followed in the Swedish tax-funded healthcare system. Therefore, we conducted a community-based survey study and analyzed the answers from parents of autistic children and autistic adolescents and adults to examine, as a primary aim, the access and utilization of CGT after ASD diagnosis. Furthermore, we analyzed the characteristics of the participants referred to CGT and whether these characteristics were following the existing guidelines. We also report the participants’ opinions on how they experienced the CGT process. As a secondary aim, we wanted to examine the awareness for CGT in ASD in Sweden in both respondent groups and gathered information needs about CGT. The results from this study can help the autism community in Sweden to be involved in modifying the guidelines for CGT after ASD diagnosis and help the healthcare section monitor the procedures across the country.

## Methods

### Survey design

We conducted two online questionnaires to survey opinions and experiences regarding genetic etiology, genetic testing, information needs, and accessibility for CGT after ASD diagnosis in Sweden. One questionnaire was targeted to parents with at least one autistic child (no limitation was set regarding the age of the child), and the other questionnaire targeted autistic adolescents (from 15 years) and adults. The reason for including autistic adolescents from 15 years of age was that according to Swedish law, children can consent to medical procedures and treatment before 18 years of age if regarded as mature enough to make such a decision. Furthermore, adolescents from 15 years of age should give consent when participating in a research study. Thus, 15 years were decided as a reasonable lower age limit for autistic individuals. The surveys were accessible online between 12 October and 1 December 2020. The survey and data collection were done using Survey&Report, version 4.3.10.5.

The survey for parents contained 62 questions in Swedish, and the survey for autistic adolescents and adults contained 51 questions, including demographic information about the respondent and their child, when applicable. Both questionnaires had closed and open-ended questions. Out of these, 2 questions for awareness, 18 questions for utilization of CGT, and 2 for information needs were analyzed (Supplementary Table 1 for parents and Supplementary Table 2 for adolescents/adults). These questions were designed based on a review of earlier literature and adjusted for the Swedish system (Li et al., 2016; Reiff et al., 2015; Vande Wydeven et al., 2012; Zhao et al., 2019a, 2019b). During the development of the questions, we received feedback from one parent of an autistic child, two genetic counselors, one medical doctor, and one interest organization representative. The revisions were minor and primarily focused on simplifying questions, shortening the questionnaire, and adding more explanations for the questions or concepts. The final survey questions in the Survey&Report web application were pilot tested by three other parents of autistic children before finalizing and publishing online after minor revisions.

Information regarding the purpose of the study, how the data would be used and stored was given at the beginning of the survey, and all respondents gave informed consent to start the survey. Also, only the respondents who answered yes to having an autistic child (or children), or being autistic themselves, were allowed to continue with the rest of the survey. The estimated time to complete the surveys was between 15 and 30 min. The study and the surveys were reviewed and approved by the Swedish Ethical Review Authority.

### Recruitment of respondents

Recruitment for potential respondents was done through different online and social media channels, including (1) advertising on Karolinska Institutet and Center of Neurodevelopmental Disorders at Karolinska Institutet webpages, (2) advertising on social media with help from different interest organizations for ASD, as well as from private persons working to increase NDD awareness, and (3) different support/interests groups on social media, either for parents of autistic children or autistic teenagers and adults. In addition, we also sent our advertisement by email and regular mail to different Child and Adolescent Psychiatry clinics and habilitation centers for posting in their waiting rooms.

### Data analysis

Data for both separate surveys were downloaded from the Survey&Report system and processed both using Microsoft Excel and R version 4.0.2. The demographic information is presented using descriptive statistics for the groups separately. To identify demographic differences in the group of children offered CGT either at the time of diagnosis or later with those that had not been offered, we tested the statistical differences using *χ*^2^ and Fisher’s exact tests. The figures with Sweden map were generated using Datawrapper (https://app.datawrapper.de/) and modified in Inkscape 1.1 vector graphics software.

### Community involvement

Several parents of autistic children and one interest organization representative were involved in revising the included questions and pilot testing of the survey, which was previously described under section “Survey design.”

## Results

### Demographic information

During the 7 weeks that the surveys were accessible online, 868 parents of autistic children (parent group) and 213 autistic adolescents or adults (autistic adolescent/adult group) completed the survey. Results describing autistic children are based on the answers from the parents in the parent group.

Demographic information of the respondents is presented in Supplementary Table 3. A majority of the respondents were female, with 94.7% (*n* = 822) in the parent group and 74.6% (*n* = 159) in the autistic adolescent/adult group. A majority of the parents had one autistic child (80.7%, *n* = 700). Two-thirds of the autistic children were boys (63.7%, *n* = 553, Table 1). Demographic and clinical information of the autistic children (parent group) and adolescents/adults is presented in Table 1 and Supplementary Table 3.

**Table 1.** Demographic and diagnosis information for the autistic children (surveyed in the parent group) and the autistic adolescent and adult group respondents.

Characteristics	Children with ASD (*n* = 868)	Adolescents and adults with ASD (*n* = 213)
	Number (%)	Number (%)
Gender		
Male	553 (63.7)	43 (20.2)
Female	291 (33.5)	159 (74.7)
Non-binary (uncertain or other)	24 (2.8)	10 (4.7)
Age at diagnosis (years)^a^		
0–4	176 (20.3)	n.a.
5–7	202 (23.3)	n.a.
8–10	195 (22.5)	n.a.
11–13	158 (18.2)	n.a.
14–18 (or over 18)	135 (15.6)	n.a.
Additional NDD diagnosis		
ADHD	327 (37.7)	64 (30.1)
ADD	134 (15.4)	39 (18.3)
Intellectual disability	91 (10.5)	4 (1.9)
Tourette syndrome	26 (3.0)	8 (3.8)
Dyslexia	66 (7.6)	19 (8.9)
Dyscalculia	13 (1.5)	6 (2.8)
Language disorder	143 (16.5)	0 (0.0)
Any somatic disease or disorder		
Yes	397 (45.7)	144 (67.6)
No	460 (53.0)	64 (30.1)
Comorbid psychiatric condition or problem		
Yes	516 (59.4)	185 (86.9)
No	352 (40.6)	28 (13.1)

ASD: autism spectrum disorder; NDD: neurodevelopmental disorder; ADHD: attention deficit hyperactivity disorder; ADD: attention deficit disorder; n.a.: not applicable.

a Age of diagnosis was not included in the survey for adolescents and adults.

A majority of autistic children (68.2%, *n* = 592) and autistic adolescents/adults (54.5%, *n* = 116) had at least one additional NDD diagnosis. The most common additional NDD diagnosis in both groups was attention deficit hyperactivity disorder (ADHD), reported in 37.7% (*n* = 327) of children and 30.1% of adolescents/adults (*n* = 64). Ninety-one children (10.5%) and four (1.9%) adolescents/adults had comorbid ID (Table 1). Co-occurring somatic conditions or diseases—such as epilepsies, autoimmune diseases, allergies, and gastrointestinal problems—were reported for 45.7% of the autistic children (*n* = 397) and 67.6% (*n* = 144) in the autistic adolescent/adult group (Table 1).

Almost 60% (*n* = 516, 59.4%) of the autistic children and 86.9% (*n* = 185) in the autistic adolescents/adult group were reported to have had one or more psychiatric conditions or problems (Table 1). Among autistic children, the most common conditions were sleep problems (37.1%) and anxiety disorder (35.6%), while in the autistic adolescent/adult group, depression (60.1%) and anxiety (58.2%) were the most commonly reported (Supplementary Table 4).

**Figure 1.**
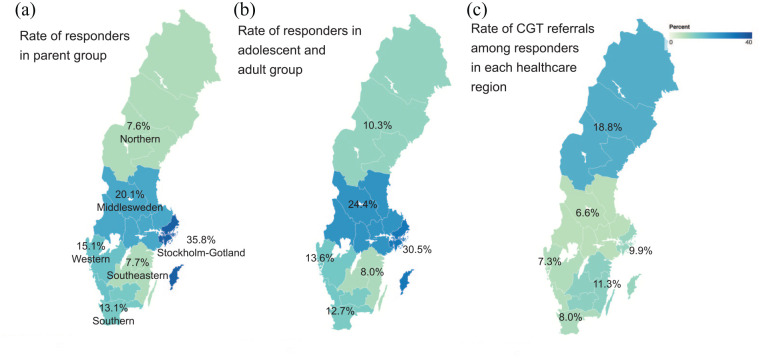
The survey response distribution for six healthcare regions in Sweden for (a) parent group and (b) autistic adolescent and adult group and (c) referral rate to CGT within each region for children in the parent group.

The distribution of the respondents by healthcare regions within Sweden is shown for the parent group in Figure 1(a) and the autistic adolescents/adult group in Figure 1(b), with the Stockholm region having the most respondents in both groups.

### Referral to CGT in the respondent groups

#### Parent group

In the parent group, 9.1% (95% confidence interval = 7.3%–11.2%, *n* = 79) reported that their child was offered a referral for CGT, either at the time of diagnosis or later. An additional 1.8% (*n* = 16) had requested a referral for genetic testing themselves, and 1.5% (*n* = 13) were not sure.

We analyzed differences in the demographic and clinical information between those children who were not offered a referral and those referred to CGT (Table 2). The autistic children offered a referral to CGT were diagnosed at a younger age category (*χ*^2^ = 82.48, degree of freedom (df) = 5, *p*-value = 2.54e−16) with the most significant difference observed for the age category between 0 and 4 years. No significant differences (*χ*^2^ = 9.32, df = 5, *p*-value = 0.10) in the rate of referrals were found between the healthcare regions. However, the referral rates ranged from 6.6% (11 out of 167 children) in Middle Sweden and 18.8% in the Northern regions (12 out of 64 children, Figure 1(c)). The autistic children offered genetic testing were more likely to have at least one other NDD diagnosis (*χ*^2^ = 6.73, *p*-value = 0.0095). The most common additional NDD in the CGT referral group was ID and language disorder. On the contrary, the autistic children within the CGT referral group were less likely to have ADHD/attention deficit disorder (ADD) or any psychiatric disorder when compared to the children who were not offered a referral (Table 2). When we investigated the demographic and diagnoses information of the respondent parents (Supplementary Table 5), the only significant difference found was that the referral group parents had a lower rate of any psychiatric disorder or condition (*χ*^2^ = 4.62, *p*-value = 0.032) (Supplementary Table 5).

**Table 2.** Differences in demographic and clinical diagnoses between autistic children either referred or not referred to clinical genetic testing based on the survey responses.

Characteristics	Referral to clinical genetic testing		*p*-value
	No (*n* = 760)	Yes (*n* = 79)	
	Number (%)	Number (%)	
Gender			**0.365**
Female	261 (34.3)	21 (26.6)	
Male	478 (62.9)	56 (70.9)	
Non-binary (uncertain or other)	21 (2.8)	2 (2.5)	
Age at diagnosis (years)			**<0.001**
0–4	118 (15.5)	44 (55.7)	
5–7	179 (23.6)	18 (22.8)	
8–10	180 (23.7)	12 (15.2)	
11–13	154 (20.3)	1 (1.3)	
14–18 (or over 18)	127 (16.7)	4 (5.1)	
Additional NDD diagnosis (any)			**0.006**
Yes	512 (67.4)	65 (82.3)	
No	248 (32.6)	14 (17.7)	
ADHD or ADD			**0.016**
Yes	416 (54.7)	32 (40.5)	
No	344 (45.3)	47 (59.5)	
Intellectual disability			**<0.001**
Yes	53 (7.0)	35 (44.3)	
No	707 (93.0)	44 (55.7)	
Language disorder			**<0.001**
Yes	107 (14.1)	28 (35.4)	
No	653 (85.9)	51 (64.6)	
Any somatic disease or condition			**0.038**
Yes	338 (44.5)	47 (59.5)	
No	412 (54.2)	31 (39.2)	
Any psychiatric condition or problem			**<0.001**
Yes	471 (62.0)	33 (41.8)	
No	289 (38.0)	46 (58.2)	

NDD: neurodevelopmental disorder; ADHD: attention deficit hyperactivity disorder; ADD: attention deficit disorder. The *p*-value is the significance.

Of those families offered a referral for CGT, 65.8% (*n* = 52) accepted and 15.2% (*n* = 12) declined. Parents accepted the referral because they wanted more information, knowledge, and understanding about their child’s condition. Additional reasons were related to family planning, including genetic syndromes as the cause of the ASD and hope for better treatments. The responding parents that declined did not record any reasons for this in the survey.

#### Autistic adolescent/adult group

In the autistic adolescent/adult group, 2.8% (95% confidence interval = 1.0%–6.0%, *n* = 6) stated that they had gotten a referral for CGT, and 3.8% (*n* = 8) requested a referral themselves. Three participants had accepted (50%) the referral, and three had declined (50%). One common reason for accepting the CGT referral was to test for Fragile X syndrome. There were no meaningful subgroup analyses for the autistic adolescent/adult group due to the low number of individuals referred to CGT.

### Reporting results after CGT in the parent group

Of the 52 families that accepted the referral, the majority got an appointment for CGT within 0–3 months (34.6%, *n* = 18) or 3–6 months (34.6%, *n* = 18). Genetic counseling before the genetic test was reported by 46.2% (*n* = 24) of the families. However, 59.6% (*n* = 31) of the parents reported that they had received enough information on why CGT was offered and its putative consequences, while 25.0% (*n* = 13) reported that they did not.

The results from the CGT were reported to the families within 6 months for 57.7% of the families (*n* = 30). Twenty-four families (46.2%) received genetic counseling together with the return of the CGT results. A few parents reported that they received the result information by phone or letter. After CGT, 40.4% of the parents reported that they received enough information to understand what the result meant for the child and the family and 40.4% (*n* = 21) reported that they did not. Most parents (59.6%, *n* = 31) were not informed or did not remember/did not know what type of CGT was done.

Ten parents (19.2%) answered that a genetic variant of clinical importance was identified, 53.8% (*n* = 28) answered that no variant was identified, and the remaining 26.9% (*n* = 14) did not want to answer or did not know. The genetic findings reported to the families included numerical and structural chromosomal abnormalities and a specified monogenic disorder/s. In three families (3/10 families, 30.0%), identifying the genetic variant led to changes in healthcare plans or interventions.

When parents were asked if the CGT results impacted their attitude toward their child and the child’s ASD diagnosis, eight parents (15.4%) answered that it had, with acceptance for the diagnosis reported commonly. An additional factor reported was the understanding that the child has a permanent condition. The results were similar when parents were asked if their understanding of ASD had changed due to the CGT, where seven (13.5%) parents answered that it had.

When parents were asked to rate the whole experience with the related healthcare during the genetic testing procedure, four parents (7.7%) answered very good, 14 (26.9%) answered good, 16 (30.8%) answered neither good nor bad, eight (15.4%) answered bad, and four (7.7%) answered very bad. Lack of information, lack of support, and lack of interventions explained why the parents were not satisfied. Nevertheless, most parents (59.6%, *n* = 31) would recommend genetic testing for ASD to other parents. When parents were asked if the result from the genetic test influenced their plans of having more children, five (9.6%) answered that it had, and 40 (76.9%) answered that it had not affected their plans.

### Awareness of CGT in the respondent groups

Respondents in both groups, who were not offered or had not requested CGT referral (*n* = 760 in the parent group and *n* = 199 in the autistic adolescent/adult group), were asked if they believed CGT for ASD is available today. Only 16.2% (*n* = 123) in the parent group and 19.6% (*n* = 39) in the autistic adolescent/adult group believed that it was. On the contrary, 34.7% (*n* = 264) in the parent group and 32.7% (*n* = 65) in the autistic adolescents/adult group believed CGT was not available. The rest were unsure or did not want to answer. Of those who believed CGT was available, 51.2% (*n* = 63) in the parent group and 25.6% (*n* = 10) in the autistic adolescents/adult group had read about it. Other information sources were indicated by 46.3% (*n* = 57) in the parent group and included knowing other families that had been offered CGT or heard about CGT during information lectures about ASD. Similarly, in the adolescent/adult group, 46.2% (*n* = 18) reported other sources.

### Information needs prior to CGT among the respondent groups

**Figure 2.**
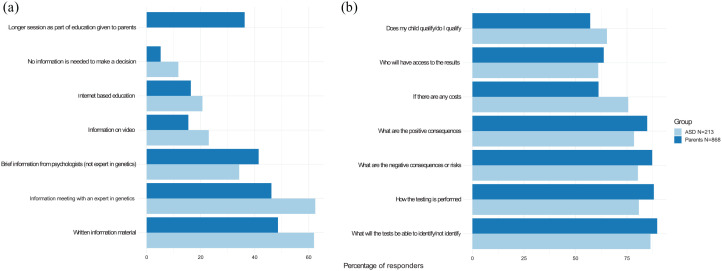
Preference for communication (a) and information needs (b) in relation to clinical genetic testing after autism spectrum disorder diagnosis among the respondent groups.

All participants were asked how they would prefer to be informed about CGT for their child (parent group) or themselves (autistic adolescent/adult group). Both groups preferred (Figure 2(a)) to be informed through written text (48.6% in the parent group and 62% in the autistic adolescent/adult group) and by an expert in genetics (46.2% in the parent group and 62.4% in the autistic adolescent/adult group). However, Internet-based education was only preferred by 16.4% in the parent group and 20.7% in the autistic adolescent/adult group. Several in the parent group commented that, most likely, their ability to take in information about CGT would be limited at the same appointment as the ASD diagnosis was made. Therefore, brief information was preferred at the time of the behavioral diagnosis, followed by information at one or several later appointments. Both groups reported that it is important to discuss the issues with a neutral person knowledgeable in genetics, psychology, and ethics.

The most important information for both groups (89.6% in the parent group and 86.4% in the autistic adolescent/adult group) was knowing what the genetic test can identify and what the limitations are (Figure 2(b)). In addition, specific information regarding the testing procedure was important to 88.0% in the parent group, where parents commented that this would help prepare the child.

## Discussion

CGT after ASD diagnosis has been recommended already for years and has been used for selected loci and genes for decades and genome-wide investigations already over a decade (Schaefer et al., 2013). Despite the recommendations and the possibilities, it is unknown for most countries to what extent CGT is utilized after ASD diagnosis.

Here, we report a relatively low referral rate (9.1%) to CGT among families with at least one autistic child based on data from a community survey of nearly 900 parents from Sweden, not limited to a single city or clinic as many of the other earlier reports on the utilization of CGT in ASD. Currently, there are no national guidelines from the Swedish National Board of Health and Welfare for CGT after ASD diagnosis; however, the recommendation from the Swedish Pediatric Association states that autistic children with ID or other developmental problems or malformations should be offered CGT. This could partly explain the lower rate of access to CGT than reported from other countries (Amiet et al., 2014; Codina-Solà et al., 2017). Indeed, we demonstrate that those autistic children who received a referral were more likely to have ID and/or language disorder than those who were not referred. However, most autistic children with comorbid ID did not get a referral to CGT, which indicates an underutilization of CGT for this group of children. Although there were no significant differences in the reported referral rates for CGT between the specific healthcare regions in Sweden, there was a range from 6.6% to almost 20%, which could indicate better access to CGT in certain regions. We also demonstrate that very few autistic adolescents and adults were offered a referral to CGT (2.8%). No other studies have investigated the referral for CGT among autistic adults as far we are aware of; however, it is an important area of research for further studies.

In Sweden and within the European Union, patients are guaranteed an appointment at a specialist clinic within 90 days after a referral. Of the 52 families in the parent group that accepted the referral, approximately one-third got an appointment for CGT within this time frame. Furthermore, the present survey shows that appropriate genetic counseling before CGT, or in conjunction with being given the result of CGT, was only available for less than half of the families. Given that genetic counseling enables parents to make an informed decision regarding testing and helps them interpret the result, it is recommended to always be a part of CGT because of the complexity of genetic contribution in ASD (Hoang et al., 2018). However, despite the lack of counseling, half of the parents were satisfied with the experience of CGT.

Since 3 out of 10 of the families that received positive results after CGT reported a change in the healthcare plan for their child, CGT promptly after the ASD diagnosis could lead to better and more individualized care. Given the small number of autistic children with a positive genetic finding, it is challenging to conclude the effect of CGT among the respondents and their families, yet it is in accordance with previous studies (Harris et al., 2020; Henderson et al., 2014). Previously reported benefits of CGT were confirmed in the open-ended answer of our survey study, including the empowerment of the families with knowledge regarding the underlying cause and providing more accurate recurrence risk counseling (Kreiman & Boles, 2020; Schaefer et al., 2013).

Finally, this study highlights the preferred ways of communicating information about CGT for parents/families and autistic individuals. Both groups preferred to be informed by written text and by an expert in genetics. Being able to ask questions was important to many participants, which highlights the importance of genetic counseling. Interestingly, Internet-based education, which was the most preferred method in previous studies (Li et al., 2016; Zhao et al., 2019b), was only preferred by approximately one-fifth of participants. A majority of respondents wanted to be informed about all suggested topics, showing a high interest in learning more about CGT.

### Limitations of the study

This study gives an overview of utilization of CGT after ASD diagnosis in Sweden, which has not previously been reported. However, the major limitation of the study is that it is based on survey answers from the community, which could include biases on the characteristics of the respondents, accuracy of the responses due to potential recall bias, or difficulty understanding the scope of the survey, and non-response bias. Based on comments, it was apparent that some participants did not understand the difference between CGT and a genetic research study, and thus, some children appear to have been included in a research study instead of having CGT. In addition, some of the participants among the autistic adolescents and adults appear to have been referred for a behavioral evaluation and not CGT. With better co-production of the survey questions with autistic individuals and additional parents of autistic children, these misunderstandings could have been avoided. Several respondents started the survey but did not finish all the questions, which could be due to many reasons, including that the survey was too long or the questions were not relevant for the respondent.

Although our recruitment for respondents was broad, statistical analysis of the daily survey responses rates indicated that most participants were recruited from online channels. Thus, there could be a possible bias toward participants interested in seeking more information or parents having children with more needs. Another limitation in the study was that the response rate of autistic adolescents and adults was much lower than parents. This could indicate that our recruitment strategy was not as successful in reaching the target group or that the group was not interested in answering the survey.

Finally, the number of individuals who received CGT was low in this study, limiting the possibility to perform more detailed analyses especially in the autistic adolescent/adult group. Furthermore, some of the children were only partially undergone the whole process of CGT and could only answer part of the questions concerning access and utilization of CGT.

## Conclusion

In conclusion, we show that the referral rates for CGT for families and autistic individuals are lower in Sweden than reported for the United States and some other European countries. However, additional investigations using, for instance, national patient registries and studies among healthcare providers for their procedures are needed for a complete picture of the current stage of CGT in Sweden for autistic individuals. Hopefully, the result of this study will facilitate access to CGT by increasing awareness about the possibility and potential benefits of CGT among parents of autistic children, autistic individuals, and healthcare providers.

## Supplemental Material

sj-pdf-1-aut-10.1177_13623613211066130 – Supplemental material for Access, utilization, and awareness for clinical genetic testing in autism spectrum disorder in Sweden: A survey study Click here for additional data file. Supplemental material, sj-pdf-1-aut-10.1177_13623613211066130 for Access, utilization, and awareness for clinical genetic testing in autism spectrum disorder in Sweden: A survey study by Anna Hellquist and Kristiina Tammimies in Autism
