# Supplementary material for: Access, utilization, and awareness for clinical genetic testing in autism spectrum disorder in Sweden: A survey study
Source: Autism. 2021 Dec 28;26(7):1795–804. doi: 10.1177/13623613211066130 (PMC9483707; doi:10.1177/13623613211066130)
Supplement: sj-pdf-1-aut-10.1177_13623613211066130 – Supplemental material for Access, utilization, and awareness for clinical genetic testing in autism spectrum disorder in Sweden: A survey study [file sj-pdf-1-aut-10.1177_13623613211066130.pdf]

Supplementary Tables

Access, Utilization, and Awareness for Clinical Genetic Testing in Autism Spectrum Disorder in Sweden – a survey study

Table of Contents

Supplementary Table 1. Overview of the survey questions to parents of autistic children..... 2

Supplementary Table 2. Overview of the survey questions to autistic adolescents (above 15 years) and adults. 7

Supplementary Table 3. Demographic information of the survey responders. 11

Supplementary Table 4. Percent of psychiatric conditions or problem reported for autistic individuals in the surveys 12

Supplementary Table 5. Differences in demographic between the parents to autistic children either referred or not referred to CGT. 13

Supplementary Table 1. Overview of the survey questions to parents of autistic children. The questions are translated to English from the Swedish version (available from the corresponding author).

1.	Does your child or children have a diagnosis of autism spectrum disorder (ASD)? [a]
	Yes, one child<br>Yes, two or more children<br>No<br>Do not wish to answer
2	What is your child's gender (with gender, we mean the gender that your child identifies with)?
	Girl<br>Boy<br>Non-binary<br>Other<br>Not sure<br>Do not wish to answer
3	How old was your child when diagnosed with ASD?
	0 - 4 years<br>5 - 7 years<br>8 - 10 years<br>11 - 13 years<br>14 - 18 years (or over 18 years)<br>Do not wish to answer
4	How old is your child today?
	0 - 4 years<br>5 - 7 years<br>8 - 10 years<br>11 - 13 years<br>14 - 18 years<br>>18 years<br>Do not wish to answer
5	In which region did you live when your child got diagnosed with ASD?
	Region Blekinge, Region Dalarna, Region Gotland, Region Gävleborg, Region Halland, Region Jämtland Härjedalen, Region Jönköping län, Region Kalmar län, Region Kronoberg, Region Norrbotten, Region Skåne, Region Stockholm, Region Sörmland, Region Uppsala, Region Värmland, Region Västerbotten, Region Västernorrland, Region Västmanland, Region Örebro län, Region Östergötland, Västra Götalandsregionen<br>Do not wish to answer
6	Does your child have any other neurodevelopmental condition (select all that apply)?
	ADHD (Attention Deficit Hyperactivity Disorder)<br>ADD (Attention Deficit Disorder)<br>Intellectual disability<br>Tourette syndrome<br>Dyslexia<br>Dyscalculia<br>Language disorder<br>No<br>Do not wish to answer
7	Does your child have any psychiatric conditions (select all that apply)?
	Depression<br>Anxiety<br>Specific phobia<br>Social phobia<br>Sleep disorder<br>Fatigue syndrome<br>Obsessive-compulsive disorder<br>Anorexia<br>Bipolar disorder<br>Psychosis<br>Schizophrenia<br>Posttraumatic stress syndrome<br>Alcohol addiction<br>Drug addiction

	Other (please specify)<br>No<br>Do not wish to answer
8	Does your child have any diseases or disorders (for example, epilepsy, allergy, diabetes, gastrointestinal problems, hearing loss, or other conditions)? Please specify what condition/s, if yes.
	Yes<br>No<br>Do not wish to answer
9	How old are you?
	15 - 18 years<br>19 - 25 years<br>26 - 35 years<br>36 - 45 years<br>46 - 55 years<br>56 - 65 years<br>> 65 years<br>Do not wish to answer
10	What is your marital status?
	Married<br>Living with a partner<br>Having a partner but not living together<br>Single<br>Other<br>Do not know/do not wish to answer
11	What is your average household income before tax (including benefits if relevant)?
	0 – 19 999 Swedish kronor<br>20 000 – 39 999 Swedish kronor<br>40 000 – 59 999 Swedish kronor<br>60 000 – 89 999 Swedish kronor<br>> 90 000 Swedish kronor<br>Do not wish to answer
12	What is your highest level of education?
	Primary and secondary school or similar<br>High school degree or similar<br>Folk high school or similar<br>Courses at university or college<br>A degree from university or college<br>Do not wish to answer
13	Are you born in Sweden?
	Yes<br>No<br>Do not wish to answer
14	What is your gender (with gender, we mean the gender that you identify with)?
	Woman<br>Man<br>Non-binary<br>Other<br>Not sure<br>Do not wish to answer
15	Do you have a diagnosis of autism spectrum disorder (ASD)?
	Yes<br>No<br>Do not wish to answer
16	Do you have any other neurodevelopmental condition (select all that apply)?
	ADHD (Attention Deficit Hyperactivity Disorder)<br>ADD (Attention Deficit Disorder)<br>Intellectual disability<br>Tourette syndrome<br>Dyslexia<br>Dyscalculia<br>Language disorder<br>No<br>Do not wish to answer
17	Do you have any psychiatric condition (select all that apply)?
	Depression<br>Anxiety

	Specific phobia<br>Social phobia<br>Sleep disorder<br>Fatigue syndrome<br>Obsessive-compulsive disorder<br>Anorexia<br>Bipolar disorder<br>Psychosis<br>Schizophrenia<br>Posttraumatic stress syndrome<br>Alcohol use disorder<br>Drug use disorder<br>Other (please specify)<br>No<br>Do not wish to answer
18	Do you have any diseases or disorders (for example, epilepsy, allergy, diabetes, gastrointestinal problems, hearing loss, or other conditions)? Please specify the condition if yes.
	Yes (open field to specify)<br>No<br>Do not wish to answer
19	My child was offered a referral to a clinical geneticist for clinical genetic testing in conjunction with being diagnosed with ASD.
	Yes, we were offered a referral for clinical genetic testing.<br>We were not offered a referral for clinical genetic testing but did ask for one ourselves.<br>We were not offered a referral for clinical genetic testing in conjunction with being diagnosed with ASD but at a later timepoint<br>No, we were not offered a referral for clinical genetic testing.<br>Don´t know/Do not wish to answer
20	I believe that clinical genetic testing is currently available for Swedish children with ASD.[b]
	Yes<br>No<br>Don’t know/Do not wish to answer
21	The reason why I believe that is.[c]
	I have read about it<br>I was informed about clinical genetic testing in conjunction with my child being diagnosed with ASD<br>Other reason (please specify).
22	We choose to ___ the referral for clinical genetic testing. Please specify why you chose to accept/decline.[d]
	Accept<br>Decline<br>Don’t know/Do not wish to answer
23	How long did it take from the date of referral for genetic testing until you and your child got an appointment for genetic testing?
	< 3 months<br>3 - 6 months<br>7 - 11 months<br>12 - 18 months<br>19 - 24 months<br>> 24 months<br>I don’t remember
24	I/we got genetic counseling at the appointment for genetic testing. With genetic counseling, we mean that a medical doctor or genetic counselor explains why genetic testing is done, what can be identified/not identified and what kind of consequences genetic testing may have for the child and family members.
	Yes<br>No<br>Don’t know/Do not wish to answer
25	I/we got sufficient information to understand why my child was offered genetic testing, what it means to do genetic testing and what consequences it may have. Please specify what additional information you needed if your answer is no.
	Yes<br>No<br>Don’t know/Do not wish to answer
26	How long did it take from the appointment for genetic testing until you received information about the results of the genetic test?
	< 3 months<br>3 - 6 months<br>7 - 11 months

	12 - 18 months<br>19 - 24 months<br>> 24 months<br>I don't remember
27	I/we got genetic counseling in conjunction with learning the results from the genetic testing. With genetic counseling, we mean that a medical doctor or genetic counselor explains why genetic testing was done, what the result was and what kind of consequences genetic testing may have for your child.
	Yes<br>No<br>Don't know/Do not wish to answer
28	I/we got sufficient information to understand what the result from the genetic testing means for my child and our family. Please specify what additional information you needed if your answer is no.
	Yes<br>No<br>Don't know/Do not wish to answer
29	Were you informed about which type/types of genetic test(s) that was done (select all that applies)?
	I was informed that they did a chromosomal analysis<br>I was informed that they did a genetic test for a specific syndrome (one or several genes)<br>I was informed that they did exome or whole genome sequencing.<br>I was informed that they did a genomic array<br>No, I was not informed regarding what kind of genetic test that was done<br>Don't know/Do not wish to answer/Don't remember
30	Did the genetic test identify a genetic variant that was connected to ASD (positive test result)?
	Yes<br>No<br>Don't know/Do not wish to answer
31	Were you informed regarding what/which type(s) of genetic variant(s) were identified (select all that apply). Please comment on how you interpret the result.[e]
	Yes, they identified a numeric chromosomal abnormality.<br>Yes, they identified a structural chromosomal abnormality.<br>Yes, they found a genetic variant (mutation) affecting one gene (monogenic disorder).<br>Yes, they found a variant of unknown significance (=VUS).<br>Other (please specify).<br>No, I was only informed that they had identified a genetic variant.<br>Don't know/Do not wish to answer/Don't remember
32	Did you, in your opinion, get enough support when you were informed about the result of the genetic test? Were you offered a follow-up visit if needed?
	Yes<br>No<br>Don't know/Do not wish to answer
33	Did the result from the genetic test lead to any change in treatment or interventions for your child? If yes, please specify which kind of treatment or intentions your child got explicitly due to the genetic test result.
	Yes<br>No<br>Don't know/Do not wish to answer
34	Did the genetic test change the way you relate to your child and his or her ASD? If yes, please specify in what way.
	Yes<br>No<br>Don't know/Do not wish to answer
35	Did the genetic test change your understanding of ASD? If yes, please specify how it has changed.
	Yes<br>No<br>Don't know/Do not wish to answer
36	How do you think that the health care given together with the genetic test has worked in general? Please specify what could have worked better, if relevant.
	Very good<br>Good<br>Neither good or bad<br>Bad<br>Very bad<br>Don't know/Do not wish to answer
37	Given your experience, would you recommend other parents a genetic test for their child with ASD? Please specify why/why not.
	Yes<br>No

	Don’t know/Do not wish to answer
38	Did the result from the genetic test affect your plans to have more children? If yes, please specify how your plans were affected.
	Yes<br>No<br>Don’t know/Do not wish to answer
39	How would you prefer to be informed in order to be able to decide on genetic testing for your child (select all that apply)? [f]
	Brief information from a psychologist (who is not an expert in genetics) in conjunction with the child being diagnosed with ASD<br>Brief information as part of an information meeting offered to parents whose children recently got diagnosed with ASD (by a person who is not an expert in genetics)<br>A longer session as part of the education given to parents whose children recently got diagnosed with ASD (by a person who is an expert in genetics)<br>Internet-based education<br>Written information material<br>Information on video<br>Privat session with a person who is an expert in genetics<br>I don’t need additional information to make such a decision<br>Don’t know/Do not wish to answer<br>Other (please specify)
40	What type of information would you like to have (select all that apply)? [f]
	If there are any costs<br>What are the positive consequences<br>What are the negative consequences or risks<br>What will the tests be able to identify/not identify<br>How the testing is done<br>Who will have access to the results<br>Does my child qualify<br>Don’t know/Do not wish to answer<br>Other (please specify)

a. If the parent answering the questionnaire has more than one child with ASD, the parent is asked to answer the following questions for the child who got diagnosed most recently
b. Only responders that answered “No, we were not offered a referral for clinical genetic testing” or “Don’t know/ Do not wish to answer” to question 19 were directed to this question.
c. Only responders that answered “Yes” to question 20 were directed to this question.
d. Only responders that answered “Yes, we were offered a referral for clinical genetic testing” or “We were not offered a referral for clinical genetic testing in conjunction with being diagnosed with ASD but at a later timepoint” to question 19 were directed to the questions 22 to 38.
e. Only responders that answered “Yes” to question 30 were directed to this question.
f. All responders answered this question.

Supplementary Table 2. Overview of the survey questions to autistic adolescents (above 15 years) and adults. The questions are translated to English from the Swedish version (available from the corresponding author).

1	Do you have a diagnosis of autism spectrum disorder (ASD)?
	Yes<br>No<br>Do not wish to answer
2	In which region did you live when you got diagnosed with ASD?
	Region Blekinge, Region Dalarna, Region Gotland, Region Gävleborg, Region Halland, Region Jämtland Härjedalen, Region Jönköping län, Region Kalmar län, Region Kronoberg, Region Norrbotten, Region Skåne, Region Stockholm, Region Sörmland, Region Uppsala, Region Värmland, Region Västerbotten, Region Västernorrland, Region Västmanland, Region Örebro län, Region Östergötland, Västra Götalandsregionen<br>Do not wish to answer
3	Do you have any other neurodevelopmental condition/s (select all that apply)?
	ADHD (Attention Deficit Hyperactivity Disorder)<br>ADD (Attention Deficit Disorder)<br>Intellectual disability<br>Tourette syndrome<br>Dyslexia<br>Dyscalculia<br>Language disorder<br>No<br>Do not wish to answer
4	Do you have any psychiatric conditions (select all that apply)?
	Depression<br>Anxiety<br>Specific phobia<br>Social phobia<br>Sleep disorder<br>Fatigue syndrome<br>Obsessive-compulsive disorder<br>Anorexia<br>Bipolar disorder<br>Psychosis<br>Schizophrenia<br>Posttraumatic stress syndrome<br>Alcohol use disorder<br>Drug use disorder<br>Other (please specify)<br>No<br>Do not wish to answer
5	Do you have any diseases or disorders (for example, epilepsy, allergy, diabetes, gastrointestinal problems, hearing loss, or other conditions)? Please specify the condition if yes.
	Yes (open field to specify)<br>No<br>Do not wish to answer
6	Do you have children?
	Yes, one child<br>Yes, two or more children<br>No<br>Do not wish to answer
7	Does your child or children have a diagnosis of autism spectrum disorder (ASD)?
	Yes, one child<br>Yes, two or more children<br>No<br>Do not wish to answer
8	Does your child have any other neurodevelopmental condition (select all that apply)?
	ADHD (Attention Deficit Hyperactivity Disorder)<br>ADD (Attention Deficit Disorder)<br>Intellectual disability<br>Tourette syndrome<br>Dyslexia

	Dyscalculia<br>Language disorder<br>No<br>Do not wish to answer
9	How old are you?
	15 - 18 years<br>19 - 25 years<br>26 - 35 years<br>36 - 45 years<br>46 - 55 years<br>56 - 65 years<br>> 65 years<br>Do not wish to answer
10	What is your marital status?
	Married<br>Living with a partner<br>Having a partner but not living together<br>Single<br>Other<br>Do not know/do not wish to answer
11	What is your average household income before tax (including benefits if relevant)?
	0 – 19 999 Swedish kronor<br>20 000 – 39 999 Swedish kronor<br>40 000 – 59 999 Swedish kronor<br>60 000 – 89 999 Swedish kronor<br>> 90 000 Swedish kronor<br>Do not wish to answer
12	What is your highest level of education?
	Primary and secondary school or similar<br>High school degree or similar<br>Folk high school or similar<br>Courses at university or college<br>A degree from university or college<br>Do not wish to answer
13	Are you born in Sweden?
	Yes<br>No<br>Do not wish to answer
14	What is your gender (with gender, we mean the gender that you identify with)?
	Woman<br>Man<br>Non-binary<br>Other<br>Not sure<br>Do not wish to answer
15	Have you (or your parents if you are/were under 18 years old) been offered, or have asked for, a genetic test for ASD?
	Yes, I (or my parents) was offered a genetic test.<br>Yes, I (or my parents) did ask for a genetic test.<br>No, I (or my parents) was not offered or have asked for a genetic test.<br>Don’t know/Do not wish to answer
16	I believe that clinical genetic testing is currently available for Swedish individuals with ASD.[a]
	Yes<br>No<br>Don’t know/Do not wish to answer
17	The reason why I believe that is.[b]
	I have read about it.<br>I was informed about clinical genetic testing in conjunction with my child being diagnosed with ASD<br>My child has done a genetic test<br>Other reason (please specify).
18	I (or my parents) choose to ___ the referral for clinical genetic testing. Please specify why you choose to accept/decline.[c]
	Accept<br>Decline<br>Don’t know/Do not wish to answer

19	How long did it take from the date of referral for genetic testing until you got an appointment for genetic testing?
	< 3 months<br>3 - 6 months<br>7 - 11 months<br>12 - 18 months<br>19 - 24 months<br>> 24 months<br>I don't remember
20	I got genetic counseling at the appointment for genetic testing. With genetic counseling, we mean that a medical doctor or genetic counselor explains why genetic testing is done, what can be identified/not identified and what kind of consequences genetic testing may have.
	Yes<br>No<br>Don't know/Do not wish to answer
21	I believe I got sufficient information to understand why I was offered genetic testing, what it means to do genetic testing and its consequences. Please specify what additional information you needed if your answer is no.
	Yes<br>No<br>Don't know/Do not wish to answer
22	How long did it take from the appointment for genetic testing until you got the results of the genetic test?
	< 3 months<br>3 - 6 months<br>7 - 11 months<br>12 - 18 months<br>19 - 24 months<br>> 24 months<br>I don't remember
23	I got genetic counseling in conjunction with learning the results of the genetic testing. With genetic counseling, we mean that a medical doctor or genetic counselor explains why genetic testing was done, what the result was, and what kind of consequences genetic testing may have.
	Yes<br>No<br>Don't know/Do not wish to answer
24	I believe I got sufficient information to understand what the result from the genetic testing means for me. Please specify what additional information you needed if your answer is no.
	Yes<br>No<br>Don't know/Do not wish to answer
25	Were you informed about which type/types of genetic test(s) that were done (select all that applies)?
	I was informed that they did a chromosomal analysis<br>I was informed that they did a genetic test for a specific syndrome (one or several genes)<br>I was informed that they did exome or whole-genome sequencing.<br>I was informed that they did a genomic array<br>No, I was not informed regarding what kind of genetic test was done<br>Don't know/Do not wish to answer/Don't remember
26	Was a genetic variant, explaining why you have ASD, identified?
	Yes<br>No<br>Don't know/Do not wish to answer
27	Were you informed regarding what/which type(s) of genetic variant(s) were identified (select all that apply). Please comment on how you interpret the result.[d]
	Yes, they identified a numeric chromosomal abnormality.<br>Yes, they identified a structural chromosomal abnormality.<br>Yes, they found a genetic variant (mutation) affecting one gene (monogenic disorder).<br>Yes, they found a variant of unknown significance (=VUS).<br>Other (please specify).<br>No, I was only informed that they had identified a genetic variant.<br>Don't know/Do not wish to answer/Don't remember
28	Did you, in your opinion, get enough support when you were informed about the result of the genetic test? Were you offered a follow-up visit if needed?
	Yes<br>No<br>Don't know/Do not wish to answer

29	Did the result from the genetic test lead to any change in treatment or interventions? If yes, please specify which kind of treatment or intentions you got explicitly due to the genetic test result.
	Yes<br>No<br>Don't know/Do not wish to answer
30	Did the genetic test change the way you relate to yourself and your diagnosis of ASD? If yes, please specify in what way.
	Yes<br>No<br>Don't know/Do not wish to answer
31	Did the genetic test change your understanding of ASD? If yes, please specify how it has changed.
	Yes<br>No<br>Don't know/Do not wish to answer
32	How do you think that the health care given together with the genetic test has worked in general? Please specify what could have worked better, if relevant.
	Very good<br>Good<br>Neither good or bad<br>Bad<br>Very bad<br>Don't know/Do not wish to answer
33	Given your experience, would you recommend others to do a genetic test for ASD? Please specify why/why not.
	Yes<br>No<br>Don't know/Do not wish to answer
34	Did the result from the genetic test affect your plans to have children, alternatively, more children? If yes, please specify how your plans were affected.
	Yes<br>No<br>Don't know/Do not wish to answer
35	How would you prefer to be informed to decide on taking a genetic test or not (select all that apply)? [e]
	Brief information from my psychologist (who is not an expert in genetics)<br>Brief information from my doctor (by a person who is not an expert in genetics)<br>Internet-based education<br>Written information material<br>Information on video<br>Privat session with a person who is an expert in genetics<br>I don't need additional information to make such a decision<br>Don't know/Do not wish to answer<br>Other (please specify)
36	What type of information would you like to have (select all that apply)? [e]
	If there are any costs<br>What are the positive consequences<br>What are the negative consequences or risks<br>What will the tests be able to identify/not identify<br>How the testing is done<br>Who will have access to the results<br>Do I qualify<br>Don't know/Do not wish to answer<br>Other (please specify)

a. Only responders that answered "No, I (or my parents) was not offered, or have asked for, a genetic test" or "Dont´t know" to question 15 were directed to this question.
b. Only responders that answered "Yes" to question 16 were directed to this question.
c. Only responders that answered "Yes, I (or my parents) was offered a genetic test" or "Yes, I (or my parents) did ask for a genetic test" to question 15 were directed to questions 18 to 34.
d. Only responders that answered "Yes" to question 26 were directed to this question.
e. All responders answered this question.

Supplementary Table 3. Demographic information of the survey responders.

	Adolescents and adults with ASD (n=213)	Parents of children with ASD (n=868)
**Characteristics**	**No. (%)**	**No. (%)**
**Gender**		
Female	159 (74.6)	822 (94.7)
**Age (years)**		
15 – 18	10 (4.7)	n.a.
19 – 25	27 (12.7)	2 (0.23)
26 – 35	70 (32.9)	92 (10.6)
36 – 45	52 (24.4)	425 (49.0)
46 – 55	32 (15.0)	310 (35.7)
> 55	22 (10.3)	38 (4.38)
**Marital status**		
Married	56 (26.3)	491 (56.6)
Living with a partner	34 (16.0)	174 (20.1)
Having a partner but not living together	13 (6.10)	24 (2.76)
Single/single parent	92 (43.2)	169 (19.5)
Other	15 (7.04)	7 (0.81)
**Household income per month (SEK before tax)**		
0 – 19 999	77 (36.2)	48 (5.53)
20 000 – 39 999	60 (28.2)	193 (22.2)
40 000 – 59 999	31 (14.6)	276 (31.8)
60 000 – 89 999	27 (12.7)	253 (29.2)
> 90 000	6 (2.82)	83 (9.56)
**Level of education**		
Primary and secondary school	40 (18.8)	38 (4.38)
High school degree	52 (24.4)	142 (16.4)
Folk high school	17 (7.98)	82 (9.45)
Courses at university or college	33 (15.5)	148 (17.1)
A degree from university or college	69 (32.4)	457 (52.7)
**Born in Sweden**		
Yes	207 (97.2)	804 (92.6)
**Child or children with ASD**		
One	45 (21.1)	700 (80.7)
Two or more	22 (10.3)	168 (19.4)
**Own diagnosis of ASD**		
Yes	213 (100)	62 (7.14)

Supplementary Table 4. Percent of psychiatric conditions or problems reported for autistic individuals in the surveys. For diagnosis or conditions with less than five individuals reported are not shown.

**Psychiatric condition or problem**	**Children with ASD (n=868)[a]**	**Adolescents and adults with ASD (n=213)**
	**No. (%)**	**No. (%)**
Depression	203 (23.4)	128 (60.1)
Anxiety	309 (35.6)	124 (58.2)
Specific phobia	32 (3.7)	19 (8.9)
Social phobia	87 (10.0)	45 (21.1)
Sleep problems	322 (37.1)	99 (46.5)
Fatigue syndrome	107 (12.3)	71 (33.3)
Obsessive-compulsive disorder	84 (9.7)	26 (12.2)
Anorexia	9 (1.0)	11 (5.2)
Bipolar disorder	5 (0.6)	8 (3.8)
Posttraumatic stress syndrome	20 (2.3)	34 (16.0)
Other psychiatric condition or problem	51 (5.9)	24 (11.3)
No	352 (40.6)	28 (13.1)

Supplementary Table 5. Differences in demographic between the parents to autistic children either referred or not referred to CGT.

	**Referral to clinical genetic testing**		**P-value[a]**
	**No (n=760)**	**Yes (n=79)**	
**Characteristics**	**No. (%)**	**No. (%)**	
**Number of children with ASD in the family**			0.184
One child	615 (80.9)	59 (74.7)	
Two or more children	145 (19.1)	20 (25.3)	
**Own ASD**			0.800
Yes	52 (6.8)	5 (6.3)	
No	704 (92.6)	74 (93.7)	
**NDD (any)**			1.000
Yes	139 (18.3)	14 (17.7)	
No	621 (81.7)	65 (82.3)	
**Disease or disorder**			0.843
Yes	377 (49.6)	41 (51.9)	
No	381 (50.1)	38 (58.1)	
**Psychiatric condition or problem**			**0.032**
Yes	419 (55.1)	33 (41.8)	
No	341 (44.9)	46 (58.2)	
**Marital status**			0.272
Married	422 (55.5)	52 (65.8)	
Living with a partner	153 (20.1)	14 (17.7)	
Having a partner but not living together	23 (3.0)	1 (1.3)	
Single parent	155 (20.4))	11 (13.9)	
Other	5 (0.7)	0 (0.0)	
**Household income per month (SEK before tax)**			0.138
0 – 19 999	43 (5.7)	3 (3.8)	
20 000 – 39 999	177 (23.3)	10 (12.7)	
40 000 – 59 999	246 (32.4)	25 (31.6)	
60 000 – 89 999	212 (27.9)	29 (36.7)	
> 90 000	70 (9.2)	9 (11.4)	
**Level of education**			0.769
Primary and secondary school	32 (4.2)	4 (5.1)	
High school degree	128 (16.8)	12 (15.2)	
Folk high school	72 (9.5)	6 (7.6)	
Courses at university or college	135 (17.8)	10 (12.7)	
A degree from university or college	392 (51.6)	47 (59.5)	

[a] Based on the χ^2 test, a significance level of 0.05 was used.
